# Impact of more variations on in‐hospital mortality among patients with confirmed COVID‐19

**DOI:** 10.1111/jch.14472

**Published:** 2022-03-21

**Authors:** Na Jia, Ping Zeng, Jun Xia, Deping Liu

**Affiliations:** ^1^ Department of Cardiology Beijing Hospital National Center of Gerontology Institute of Geriatric Medicine Chinese Academy of Medical Sciences Beijing China; ^2^ Department of Epidemiology The Key Laboratory of Geriatrics Beijing Institute of Geriatrics Beijing Hospital National Center of Gerontology Beijing China; ^3^ Institute of Geriatric Medicine Chinese Academy of Medical Sciences Beijing China; ^4^ Nottingham China Health Institute The University of Nottingham Ningbo China

Thanks for Aftab and co‐authors’ feedback on this systematic review. Three questions were raised in their letter. We tried to respond to the best of our knowledge, in this letter.

Question 1. In‐hospital mortality rates decreased in the US over 2020. A retrospective analysis during this time period would be strengthened by accounting for the variation.

Response: Several factors may influence mortality rate, for example, optimized treatment plan, viral toxicity attenuation and so on. Most of the included studies in our systematic review were carried out in early stage of the global pandemic (i.e., up to June 2020) in hospital setting with inpatients, thus we were unable to make inference across time. Patients in those earlier studies experienced similar level of improvement despite of the status of angiotensin converting enzyme inhibitors (ACEI)/angiotensin receptor blockers (ARB) use. Nevertheless, following Aftab's feedback and example of differential findings across time in US studies, we double checked data from the three US studies included in our review to examine if such trend was present. Those three studies were conducted in April, May, and June 2020, and none showed clear difference between groups. There might have been a change to the above finding in studies conducted beyond this period, with consideration to virus mutation and the use of vaccination, hence we urge future reviews to explore the time factor.

Question 2. Socioeconomic and sociocultural variations in mortality should be considered.

Response: We suggest to look at the impact of this from the angles of in‐hospital mortality and mortality in community settings. We agree with Aftab and co‐authors that in‐hospital mortality may be impacted by socioeconomic and sociocultural factors. The in‐hospital mortality rate varied between 7% and 30%^1^, ^2^, ^3^, ^4^, ^5^ across different countries. Tereshchenko's study^6^ published in 2021 indicated unhealth life style, poverty, lack of health insurance, poor education may influence the application of ACEI/ARB. Compared with less‐developed nations, patients in developed nations may have higher chance of receiving ACEI/ARB drugs, hospitalization, more aggressive treatment and vaccination. However, earlier Covid‐19 studies at the start of the global pandemic lacked reporting or relevant stratification by these socioeconomic factors. In absence of individual data or subgroup data on these domains, we were unable to carry out further analysis. But we agree this is an important issue which should be addressed in further analysis. We appeal to clinical researchers to record and report such information in future clinical studies.

While we also agree with Aftab and co‐authors that socioeconomic and sociocultural variation may alter the risk of mortality in general (beyond hospital setting), this is an epidemiological investigation which is beyond the scope of our study (which focuses on in‐hospital outcomes).

Question 3. Continuation or discontinuation of the ACEI/ARB after discharge from the hospital was not controlled.

Response: Aftab and co‐authors highlighted an important issue. This was taken into account in our protocol design. We planned to do subgroup analysis on continuation versus discontinuation of ACEI/ARB, but we had only a single randomized control trial (NCT04338009) included in the review, hence were unable to carry out subgroup analysis. The single RCT showed that the mortality rate between discontinuation and continuation of ACEI/ARB was 12.99% versus 14.67%, which is higher than the finding from the Brazil study.^7^ This could be resulted by varied disease severity and existing comorbidities, but we are not sure.

In conclusion, we thank Aftab and co‐authors for their thought‐provoking feedback. In general, we agree that these are important confounding issues which begs for further investigation, should data becomes available in future updates.

## CONFLICT OF INTEREST

The authors have no conflict of interest.
